# Deaths Due to Mixed Infections with *Passalurus ambiguus*, *Eimeria* spp. and *Cyniclomyces guttulatus* in an Industrial Rabbit Farm in Greece

**DOI:** 10.3390/pathogens10060756

**Published:** 2021-06-15

**Authors:** Georgios Sioutas, Konstantinos Evangelou, Antonios Vlachavas, Elias Papadopoulos

**Affiliations:** Laboratory of Parasitology and Parasitic Diseases, Faculty of Health Sciences, School of Veterinary Medicine, Aristotle University of Thessaloniki, 54124 Thessaloniki, Greece; Gsioutas@vet.auth.gr (G.S.); info@diagnovet.gr (K.E.); avlachav@vet.auth.gr (A.V.)

**Keywords:** *Passalurus ambiguus*, *Eimeria* spp., *Cyniclomyces guttulatus*, Industrial rabbit farm, coccidiosis, diarrhoea, deaths, fenbendazole, oregano

## Abstract

Domestic rabbits are commercially farmed for their meat whilst gastrointestinal diseases can hinder their production. *Passalurus* *ambiguus* and *Eimeria* spp. are two common rabbit intestinal parasites that can cause diarrhoea, among other symptoms, and in severe cases, death. *C. guttulatus* is a commensal yeast of the rabbits’ stomach that is considered apathogenic but can worsen symptoms in rabbits suffering from coccidiosis. In the present case report, we describe an outbreak of deaths in three different age groups (A: lactating does, B: 58 days old and C: 80 days old) in an industrial rabbit farm in Greece. Symptoms included depression, diarrhoea, inappetence, weight loss, dehydration and ruffled furs. Using a faecal flotation technique, sick rabbits were found to be moderately to heavily infected with *P. ambiguus, Eimeria* spp. and *C. guttulatus*. Treatment with fenbendazole and oregano oil combined with hygiene control measures successfully controlled the infections and resolved clinical symptoms. A faecal flotation method or other reliable diagnostic technique should be used regularly in industrial rabbit farms to screen for gastrointestinal parasitic infections. Early diagnosis and control will help to maintain production levels and, therefore, limit financial losses for the farmer while ensuring animal welfare.

## 1. Introduction

Domestic rabbits belonging to the species *Oryctolagus cuniculus* are social animals originating from European rabbits [1]. Pet rabbits have increased in recent years [1], while they continue to be used as laboratory animals in experiments [2]. Commercially, rabbits are farmed for their meat [3] and fur, although the latter production is of nominal significance in the European Union [4]. Rabbit meat intended for human consumption must first pass a thorough sanitary, safety and quality inspection by licensed veterinarians [5]. Rabbit breeds used for meat production must have a high growth rate and a high feed conversion ratio [1]. One such hybrid with good production performance commonly used in Europe is the HyCole [6,7]. Industrial rabbit farming represents a robust but often neglected agricultural enterprise in Greece that began developing around 1970 [8]. According to official data from FAOSTAT (Food and Agriculture Organization of the United Nations), in 2018, there were about 1,5 million rabbits farmed in Greece, producing approximately 2700 tons of rabbit meat, and these numbers have been steadily declining through the years [9]. With the average domestic consumption being about 7000 tons, the resulting production deficit is usually covered by imports of rabbit meat, mainly from Italy. Five large commercial farms are currently operating in various parts of the country [8]. It is well known that rabbits intended for meat consumption can harbour many different endoparasites, including *Passalurus ambiguus* [10] and *Eimeria* spp. [11].

*Passalurus ambiguus* (Rudolphi, 1819), known as the rabbit pinworm, belongs to the Oxyuroidea family and is one of the most common rabbit and hare nematodes with a worldwide distribution [12]. It has a direct life cycle [1]. Rabbits shed sticky [13], embryonated eggs [14] with their soft or hard faeces [1], which are immediately infective, each one containing a stage three larva [15]. *P. ambiguus* eggs are ovoid [1], have a light brown colour [15] and double-shelled thin walls [14], which are asymmetric [15] and marginally flat on one side. They have a size of 95–103 × 43 μm [12] and a polar plug at one end [15]. Rabbits get infected after ingesting infective eggs, usually when grooming, eating their faeces [13], or through contaminated food, water and other materials [16]. Adult pinworms usually parasitise the caecum and colon of rabbits [12]. Females lay their eggs on the perineum of the rabbit, where the eggs sometimes stick [13]. The prepatent period can range from 55–60 days [15], and the incubation period is typically 18 days [1].

Coccidiosis is an ubiquitous [12] common disease of rabbits bred in industrial farms. The etiologic agent is the protozoan parasite *Eimeria* spp., which has a direct life cycle. *Eimeria* spp. parasites have a high host specificity, and rabbits can get infected by at least 14 different *Eimeria* species. Thirteen of them infect different target sites and depths of the mucosa of the small intestine, caecum or colon, causing intestinal coccidiosis. The last species, *Eimeria stiedae*, can be found in the endothelial cells of the bile ducts, causing hepatic coccidiosis.

Rabbits shed unsporulated *Eimeria* oocysts with their faeces that need to sporulate in order to become infective. Sporulation can take 1–4 days, depending on the species, and is affected by the humidity [12] and temperature of the environment [17]. Oocysts are very resistant and can survive for several years under different environmental conditions but cannot tolerate desiccation [18]. Transmission occurs through the faecal-oral route. Rabbits get infected when ingesting sporulated oocysts from food or water contaminated with faeces [19]. They do not get auto-infected when consuming their soft faeces (cecotrophy) because those faeces contain unsporulated *Eimeria* oocysts, which are not infective [11]. Consequently, oocysts are released inside the intestinal lumen [1] and excreted with faeces. *E. stiedai* has also been successfully transmitted with blood transfusions from infected to healthy rabbits [20], intramuscularly and intraperitoneally [21]. The prepatent period varies from 5–10 days for intestinal coccidiosis [5] and 14–18 days for hepatic coccidiosis [19].

*Cyniclomyces guttulatus* is a budding, sporogenous [1], symbiotic ascomycetous yeast and part of the normal microflora of the gastrointestinal tract of different animals, including domestic and wild rabbits [22,23,24,25,26], hares, chinchillas and guinea pigs [23,24]. It was first described in 1853 as *Cryptococcus guttulatus* and was renamed and reclassified several times before acquiring its final name as *Cyniclomyces guttulatus* in 1971 [27].

Rabbits usually get infected through the faecal-oral route when eating their faeces or other contaminated plants [28]. Following ingestion, *C. guttulatus* reaches the uninfected host’s stomach and colonises it if the normal flora allows it [28]. Cecotrophy helps sustain the yeast population through continuous auto-infections [23,24]. After colonisation, *C. guttulatus* multiplies rapidly, and the numerous vegetative cells mix with the stomach content. Vegetative cells pass through the gastrointestinal tract and are excreted with the faeces, while some of them form ascospores in the large intestine [24] or outside the rabbit [23]. Usually, vegetative cells and not spores are found in rabbit faeces [29].

## 2. Case Presentation

### 2.1. Clinical Description and Colony History

An outbreak of deaths was recorded at the start of February 2021 in one of Greece’s largest industrial rabbit farms in the area of Epirus (Ioannina). Rabbits belonged to the HyCole hybrid. The commercial farm consisted of 3000 does in total reared in stainless-steel cages. The farm consisted of different buildings. Each building included six separate rooms with animals of the same age. Cages had a sufficient size allowing rabbits to express normal behaviour. Cage floors were made of wire mesh and cleaned with pressurised water once in two weeks, while the beddings consisted of good-quality hay. Considering as Day 0 the day of kindling, rabbits were bred as follows. Weaning took place on Day 41. Consequently, after Day 75, rabbit slaughtering began and lasted until Day 90–100. All rabbits had ad libitum access to food. Kittens fed on the doe’s milk from Day 0–41. They were also provided with a commercial starter (creep) diet from Day 7–22. Afterwards, from Day 22–52, they were given a grower diet and finally, from Day 52 until slaughtering, they were fed a finisher diet. All does were fed a standard lactation diet. No coccidiostats or anthelmintics were routinely administered to the rabbits.

The affected rabbits belonged to both sexes of three different age groups. Group A: lactating does, Group B: 58 days old young rabbits and Group C: 80 days old young rabbits. Each age group was housed in various rooms in separate buildings. Only certain rooms of the buildings included rabbits with clinical signs (most symptoms were common with some variances), while the rest of the rooms housed clinically healthy animals. More specifically, in Group A one room out of six was affected, in Group B one out of six and in Group C two out of six rooms.

Group A: lactating does were in their second productive cycle and housed with their 7 days old kittens, which were symptomless. Main clinical symptoms included depression, diarrhoea, inappetence, weight loss, dehydration and ruffled furs. The faeces excreted were dark, soft, watery and foul-smelling. The does experienced the least severe symptoms and recorded only a few deaths compared to other age groups.

Group B: 58 days old young rabbits exhibited depression, diarrhoea, inappetence, reduced weight gain, dehydration and ruffled furs. The faeces excreted were dark, soft, watery and foul-smelling.

**Figure 1:**
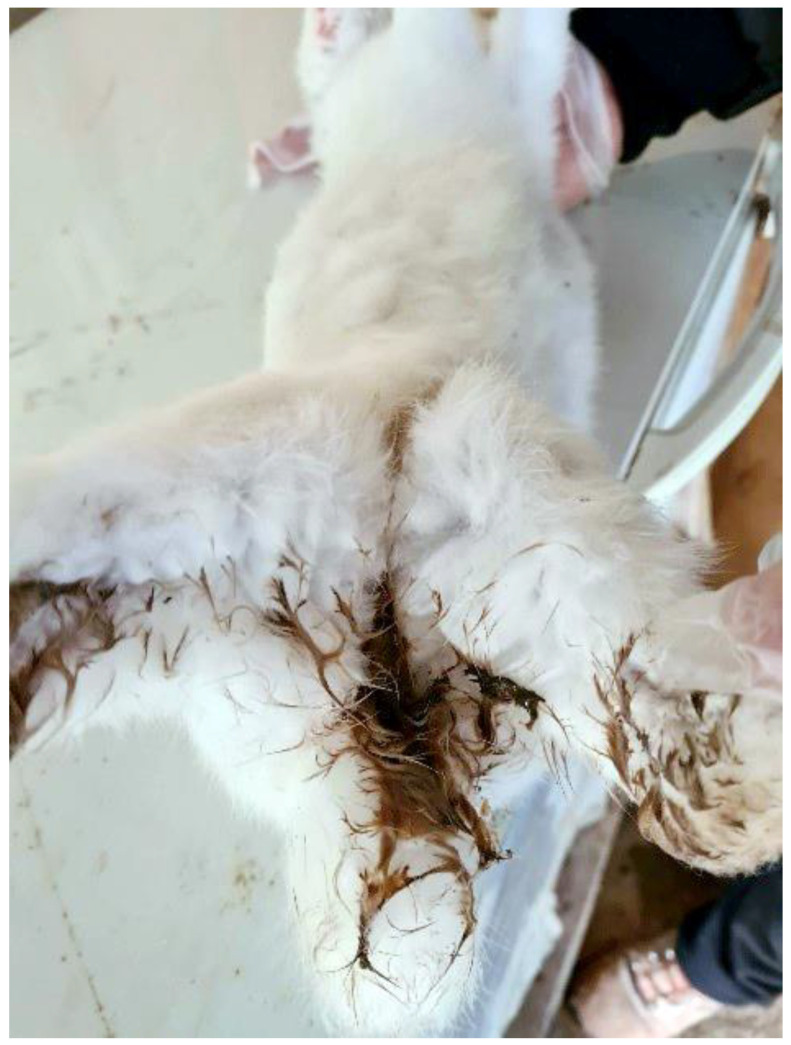
Dead rabbit with a dirty perianal area due to heavy diarrhoea.

Group C: 80 days old young rabbits exhibited depression, diarrhoea, inappetence, reduced weight gain, dehydration, abdominal distention and ruffled furs. The faeces excreted were dark, soft, watery, foul-smelling, occasionally bloody and mucoid. As a result, rabbits had a dirty perianal area (Figure 1) and a higher mortality rate than Groups A and B.

According to the farm records, the disease was acute, with clinical signs evolving rapidly, resulting in the deaths of the affected rabbits. The farm veterinarian contacted the Laboratory of Parasitology and Parasitic Diseases, School of Veterinary Medicine, Aristotle University of Thessaloniki, Greece, and was instructed to provide faecal samples from each group. The samples were collected over 24 h from the animals of all groups and stored in separate urine collection containers until arrival at the Laboratory. They were transported in less than 12 h after collection.

### 2.2. Microscopic Identification of the Parasites

We performed a faecal flotation technique on representative pooled samples from 100 animals of each group (from affected rooms). Also, similar pooled samples were examined from 100 healthy animals from each group (originating from rooms that had not any sign of infections). The methodology was as follows. Three grams of faecal material from each container were weighted and placed into separate 15 mL glass conical centrifuge tubes. The tubes were then filled with tap water, and the faecal samples were homogenised with a wooden applicator stick. Faecal samples were filtered through a wire mesh (aperture 250 μm) to eliminate coarse faecal debris, and the faecal suspension was transferred into new tubes. The tubes were then centrifuged at 1500 RPM (Revolutions Per Minute) for 3 min. After centrifuging, the supernatant was discarded, and the tubes were half-filled with a 33% zinc sulfate solution (Specific Gravity: 1.34). The faecal residues were resuspended with a wooden applicator stick, and the tubes were then fully filled with zinc sulfate until a meniscus was formed. An 18 mm x 18 mm square coverslip was carefully placed on top of the tubes before they were centrifuged again at 1000 RPM for 3 min. Subsequently, the coverslips were removed and transferred on separate microscope slides for examination under a light optical microscope (Olympus, CX21 Microscope) at 100× and 400× magnification. Faecal flotation techniques are not quantitative like the McMaster technique, but they can be used for the semi-quantitative assessment of the number of eggs, oocysts or yeast cells [11,30]. Criteria used to quantitate the infections were according to Percy et al. [11]: 1–100 oocysts/eggs/yeast cells per coverslip on a microscopic slide =+; 100–300 oocysts/eggs/yeast cells per coverslip =++; >300 oocysts/eggs/yeast cells per coverslip = + + +.

Optical microscopy revealed the following:

Group A: In sick animals, heavy infection (+++) with *C. guttulatus* vegetative cells and moderate infection (++) with *P. ambiguus* eggs were found, while in healthy animals from the unaffected rooms only a small number (+) of *C. guttulatus* vegetative cells was recorded.

Group B: In sick animals, heavy infection (+++) with *C. guttulatus* vegetative cells and heavy infection (+++) with *P. ambiguus* eggs were found, while in healthy animals only a small number (+) of *C. guttulatus* vegetative cells was recorded.

Group C: In sick animals, heavy infection (+++) with *C. guttulatus* vegetative cells, moderate infection (++) with *P. ambiguus* eggs and heavy infection (+++) with different *Eimeria* spp. oocysts were found, while in healthy animals only a small number (+) of *C. guttulatus* vegetative cells was recorded.

**Figure 2:**
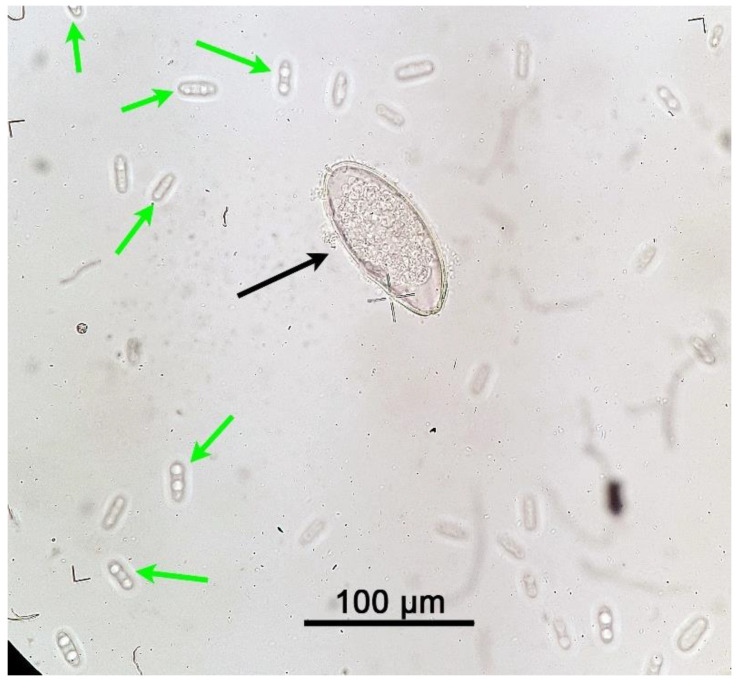
*Passalurus ambiguus* egg (black arrow) and *Cyniclomyces guttulatus* vegetative cells (green arrows) under an optical microscope at 400× magnification. Rabbit faeces were examined after using a faecal flotation technique on pooled samples from Group A (lactating does), Group B (58 days old) and Group C (80 days old).

**Figure 3:**
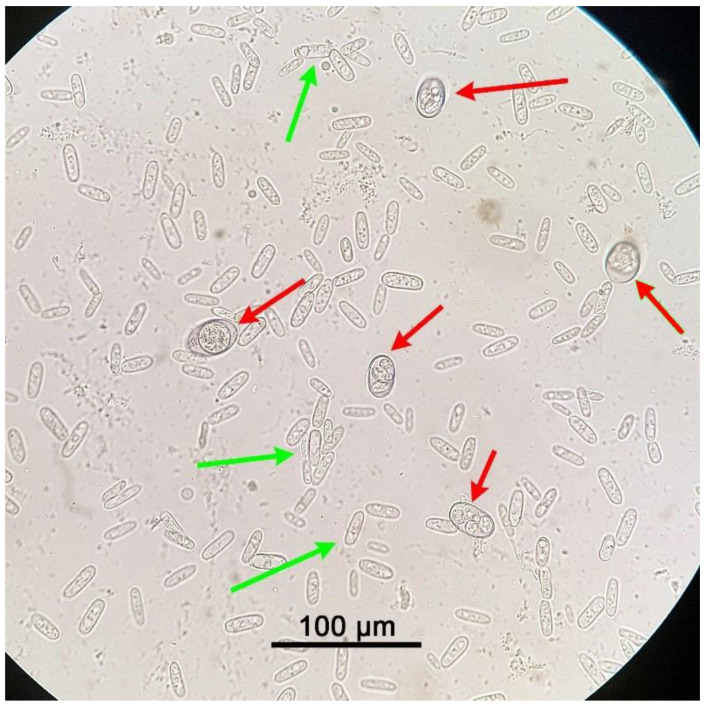
*Eimeria* spp. oocysts (red arrows) and *Cyniclomyces guttulatus* vegetative cells (green arrows under an optical microscope at 400× magnification. Rabbit faeces were examined after using a faecal flotation technique on pooled samples from Group C (80 days old).

**Figure 4:**
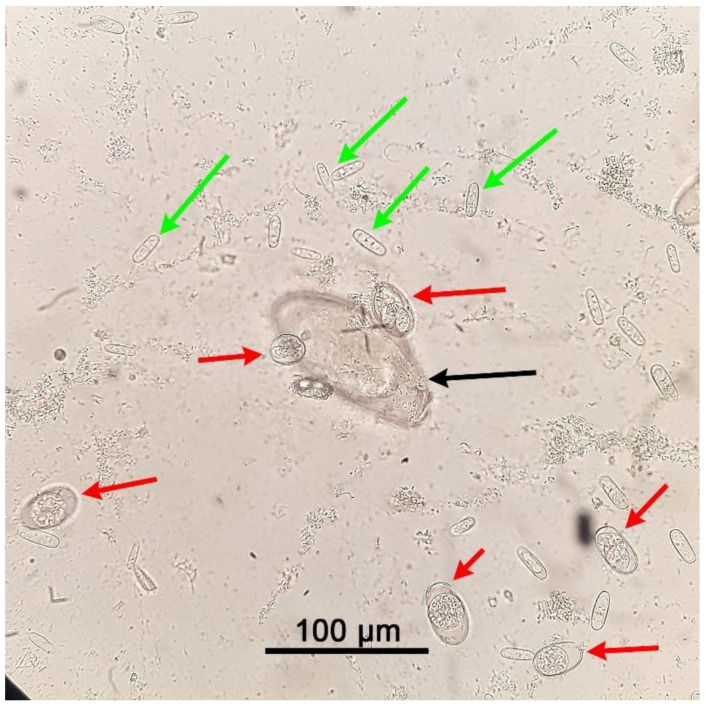
*Passalurus ambiguus* egg (black arrow), *Eimeria* spp. oocysts (red arrows), and *Cyniclomyces guttulatus* vegetative cells (green arrows) under an optical microscope at 400× magnification. Rabbit faeces were examined after using a faecal flotation technique on pooled samples from Group C (80 days old).

Results of the parasitological analysis of affected animals are summarised in Table 1. *C. guttulatus* vegetative cells can be seen in Figure 2, Figure 3 and Figure 4, *P. ambiguus* eggs in Figure 2 and Figure 4 and *Eimeria* spp. oocysts in Figure 3 and Figure 4.

### 2.3. Treatment and Control of the Parasitic and Yeast Infection

After identifying *P. ambiguus*, *Eimeria* spp. and *C. guttulatus*, the following treatments were administered to all rabbits from the affected rooms:

Group A: *P. ambiguus* was treated with a commercial suspension containing fenbendazole (Gallifen^®^ Oral Suspension 200 mg/mL, Nuevo S.A., Greece) administered orally at a dose rate of 20 mg/kg once and repeated in 14 days. No other chemical treatment was applied.

Group B: *P. ambiguus* was treated as in Group A. No other chemical treatment was applied.

Group C: *P. ambiguus* was treated as in Group A. *Eimeria* spp. was treated with a commercial solution containing oregano (Orego-Stim^®^, Anpario Medical Products, Nottinghamshire U.K.) at a dose rate of 500 mL/tonne of drinking water for 7 consecutive days. No other treatment was applied.

The specific products mentioned above are not licensed for use in rabbits. The use of unlicensed drugs is a profound problem for the country’s rabbit industry because most treatments of common diseases require the off-label use of drugs licensed for other animal species. Fenbendazole dose was based on experts’ recommendations in treating passaluriosis [31,32]. Treatment for *C. guttulatus* requires the antifungal drug nystatin, and currently, there is no commercial veterinary product in Greece containing nystatin. Furthermore, *C. guttulatus* is an opportunistic pathogen, and it causes more severe symptoms in rabbits suffering from coccidiosis. On its own, *C. guttulatus* does not seem to be pathogenic [33]. Based on this evidence, no chemical treatment for *C. guttulatus* was recommended, but a moderate carbohydrate restriction and dietary fibre increase in all animals of the affected rooms. Additional control measures included removing infected faeces and weekly cleaning of cages, particularly cage floors and feed boxes. Facility workers were instructed to wear gloves when handling rabbits and change overalls and shoes between different rooms. Not any treatment was administered to healthy animals housed in the unaffected rooms.

Following treatment, almost all rabbits showed clinical improvement and all the symptoms resolved within 15 days. A follow-up examination of faeces using the same sampling method and flotation technique described above 16 days after treatment revealed no helminth eggs. A small number (+) of *Eimeria* spp. oocysts were still present in animals of Group C and only a *few C. guttulatus* vegetative cells in all rabbits, that were of no clinical importance (similar to the animals of the unaffected rooms). The farm veterinarian reported no new diarrhoea cases or deaths. All rabbits tolerated the treatments well, and no side effects were reported. As a result, the farm owner could finally send rabbits from Group C to the abattoir and limit his financial losses.

## 3. Discussion

The present case is a good example of a classic disease in rabbits including three common pathogens (coccidia, pinworms and a yeast), though it has never been linked with deaths of rabbits in Greece. Gastrointestinal disorders that cause diarrhoea in rabbits (i.e., coccidiosis) can lead to high mortality rates [34], which in some cases reach 70% [35]. Simultaneously, intensive rabbit farming has led to increased transmission of parasites with a direct life cycle like *P. ambiguus* and *Eimeria* spp. [10,36].

*P. ambiguus* infections can impact production performance in young rabbits and does [37]. Auto-infections with *P. ambiguus* can lead to the parasite’s permanent circulation in industrial rabbit farms if no control measures are taken [38]. Lactating does in Group A might have infected their offspring (7 days old kittens) through contaminated bedding or other materials. Considering that the prepatent period for *P. ambiguus* is 55 days, we suggested a faecal examination to be performed on the kittens after weaning, when they reach approximately 67 days of age. Similarly, rabbits in Group B (58 days old) probably acquired the infection from their mothers, and we proposed a faecal examination on those does as well.

Most infections are usually asymptomatic, even with a high parasitic burden [12]. However, young rabbits or rabbits that live in colonies, such as intensive rabbit farms, have a higher risk for developing clinical signs [12,15]. Particularly in young rabbits, passaluriosis is thought to aid in the enteritis complex [1]. Symptoms may include diarrhoea, weight loss, neurological signs, like thumping with the hind feet [15], and even death [37]. The resulting weight loss can also reduce fertility in female rabbits [38], which was not reported in the current farm.

Diagnosis of passaluriosis is achieved with a faecal flotation method, i.e., using a ZnSO4 solution [1]. The transparent adhesive tape test (“Scotch tape” test), although less reliable, can also be used [37]. When comparing diagnostic methods, Rinaldi et al. found that the FLOTAC technique has higher sensitivity than the cellophane tape test and the McMaster technique [10]. Other techniques include sedimentation and a direct faecal smear [39]. Rabbit faeces should be collected during the afternoon and night hours to increase the likelihood of detecting *P. ambiguus* [10], and that is one of the reasons we suggested faeces were collected over the course of 24 h.

In the case treatment is needed, fenbendazole at a dose of 15–20 mg/kg mixed with feed for five consecutive days has proved successful [37]. Piperazine [1] or thiabendazole can also be administered, but not ivermectin which is not effective in clearing the infection [32]. Additional control measures include removing infected faeces by disinfecting rabbit cages and rotational grazing, especially for younger rabbits [1]. Following treatment, the prognosis is good, but complete elimination of the parasite and prevention of re-infections is troublesome because the eggs can tolerate high temperatures and a plethora of disinfectants [32]. In our case, thorough cleaning of cages, strict biosecurity measures to avoid contaminations (i.e., gloves, different overalls, and shoes between different houses) and removing infected faeces combined with fenbendazole successfully treated passaluriosis and prevented re-infections.

Infection rates in rabbit farms can reach up to 31% [15]. The number of *P. ambiguus* eggs shed depends on the season and age of rabbits, but not on the sex or spaying of the female rabbits [40].

Intestinal coccidiosis is quite common in industrial rabbit farms, especially in young rabbits after weaning. Infections are usually asymptomatic, and mixed infections with different *Eimeria* species are frequently observed [11], as in our case. Coccidiosis is one of the main reasons for meat rejection in slaughter rabbits, leading to substantial economic losses for the producer. It can also reduce profits through diarrhoea, weight loss, reduced growth rate and feed conversion ratio, and mortality [5,41]

Some species, like *E. flavescens* and *E. intestinalis*, are highly pathogenic, affecting the intestinal crypts. Others are moderately pathogenic, like *E. magna* and *E. media*, while some, such as *E. perforans* and *E. coecicola*, rarely cause disease [12]. As a result, some coccidia infections can be subclinical and remain undetected [42]. Symptoms are usually seen in weaned rabbits [12]. These typically include watery, dark, foul-smelling and occasionally bloody diarrhoea, anorexia and severe weight loss, dehydration and a messy fur coat [43,44]. In rabbits, signs of diarrhoea are a dirty perineal area and liquid or unusually soft faeces with the presence of mucus [45]. Additional less common symptoms are nervous signs (uncoordinated movement of the limbs), abdominal distention, stunt growth [45] and mortality [45,46]. Coccidiosis with *E*. *perforans* has also been linked to intussusception leading to death in a rabbit [47]. *E. stiedai* infections can also cause ascites or liver and gall-bladder enlargement, jaundice, constipation and fatal liver failure, particularly in young rabbits 60–90 days old [19]. Furthermore, coccidiosis is considered the leading cause of the enteritis complex, especially in young rabbits [11]. All these symptoms combined and the cost of coccidiosis control can lead to significant financial losses for the producer [46]. It is worth noting that mixed infections with different *Eimeria* species, like in the current case, are not uncommon [11,42,45,48,49,50], with the most frequents species encountered being *E. media, E. magna* and *E. perforans* [19]. However, after the first infection, rabbits seem to acquire protective immunity against the specific *Eimeria* species they were infected with [18,44,51]. Infection with one species does not provide cross-immunity to other *Eimeria* spp. [1], and immunity is not transferred from the mother to the offspring [19].

Diagnosis can be achieved by post-mortem examination of the intestinal or hepatic lesions. The different species can be distinguished by their location and lesions caused. Other methods include direct faecal smears, isolation of preferably sporulated oocysts from faeces with a faecal flotation method, the McMaster technique, or other centrifugation techniques [19,41]. Species differentiation is based on examining sporulated oocyst size and morphology under a light microscope [12]. Morphological identification is often difficult because many *Eimeria* spp. sizes and characteristics overlap, requiring the use of additional molecular tools.

Chemoprophylaxis prevents disease occurrence in healthy rabbits while treatment is used to manage disease outbreaks [12]. Chemoprevention can be achieved with the addition of certain compounds in the rabbit feed. These include monensin [52], robenidine, a coccidiostat and coccidiocidal for intestinal coccidiosis, and clopidol, a coccidiostat used around exposure for both intestinal and hepatic coccidiosis [53]. Regarding treatment, there are other drug compounds used to fight off intestinal and hepatic rabbit coccidiosis that are mainly administered via drinking water. These are salinomycin (narasin) [54], lasalocid, maduramycin [55], toltrazuril [56,57], diclazuril [58], and sulfonamides (i.e., sulfachloropyrazine) [43]. Ivermectin has also proved partially effective under experimental conditions [59]. Other control measures required are regular cleaning of wired cages, especially cage floors and feed boxes [11,12]. Pest control should be implemented if possible, and young infected rabbits should be isolated from healthy adults [19].

Alternative control measures include the administration of non-chemical drugs. There are many herbal formulations, some of which contain oregano, that can be used to control coccidiosis. Oregano’s anticoccidial activity has been demonstrated in broiler chickens [60]. Its efficacy has also been proven against rabbit *Eimeria* spp., particularly when administered prophylactically [61,62,63]. Other formulations include garlic [64,65] which has also proven effective against hepatic coccidiosis, mainly when used prophylactically [61,64]. The combination of garlic and oregano added in rabbit feed has also been shown to enhance meat quality characteristics, with zero withdrawal period [63]. Similarly, rabbit diets can be enriched with prebiotics [66] or with sanfoin [67] for coccidiosis prevention. Lactoferricin [68], heterocyclic thione derivatives [69], artemisinin liquid extract, different oils [70,71], *Salix babylonica* [72] and a commercial herbal extract [73] also showed promising results in rabbits infected with *Eimeria* spp. However, further research is needed for most of them in order to confirm their effectiveness. Likewise, vaccine development against *E. stiedae*, *E*. *magna, E. intestinalis* and *E. media* represents a future prospect for the prevention of hepatic and intestinal coccidiosis [74,75,76,77]. In our case, the administration of the commercial solution containing oregano in the drinking water at the dose rate mentioned above, successfully boosted host immunity. This resulted in the elimination of coccidiosis symptoms and significant reduction of oocyst shedding in rabbits from Group C (<100 oocysts per coverslip) with no residues.

Coccidia prevalence in industrial rabbit farms can reach up to 78% [5] and up to 100% in young rabbits [78]. Predisposing factors include a humid environment [79] and poor sanitary conditions that can lead to massive environmental contaminations [1]. Adult rabbits are typically asymptomatic carriers but can have coccidiosis lesions in their carcass [5]. Moreover, adults and especially does, that shed more oocysts during the periparturient period can act as a natural reservoir of infection for kittens [80]. Kittens are more susceptible to coccidia infections than adults [36,46], particularly right after weaning [81], which is a stressful situation for them [80]. In contrast, suckling kittens during their first 20 days of age are rarely infected with coccidia [82]. One study found a higher intensity and extensity of coccidia invasion in rabbits kept in groups, like the rabbits in the present case, compared to rabbits housed individually [83]. Coccidiosis outbreaks have also been associated with an increase in *Escherichia coli* [84], and other intestinal bacterial infections [85] in rabbits.

Regarding *C. guttulatus*, rabbit coccidiosis seems to increase the yeasts’ numbers [86]. Usually, *C. guttulatus* is considered apathogenic, and in the absence of other pathogens, does not cause clinical signs or lesions in rabbits, even in large numbers [25,26,33,86]. On the contrary, it can be considered a probiotic, particularly for weaned rabbits. Specific pathogen-free rabbits inoculated with *C. guttulatus* increased their body weight gain and feed intake [33]. However, the yeast is commonly isolated with bacteria [29] and can cause more severe symptoms and lesions in rabbits suffering from gastrointestinal diseases, such as coccidiosis [33,86]. Specifically, rabbits coinfected with *C. guttulatus* and *E*. *intestinalis* had worse symptoms, such as anorexia, reduced body weight, constipation, diarrhoea and mortality, compared to rabbits infected only with *E. intestinalis*. Besides, rabbits with coinfections shed more vegetative cells but less *Eimeria* oocysts than rabbits infected only with *C. guttulatus* or *E. intestinalis*, respectively [33]. Therefore, this yeast can be considered a secondary opportunistic pathogen [30,33]. *C. guttulatus* has also been linked to acute, dark, aqueous, and foul-smelling diarrhoea in rabbits [29,87], along with apathy [87]. As in our case, healthy rabbits from unaffected rooms were also carrying low numbers of *C. guttulatus* vegetative cells which did not have any clinical implications in the absence of other intestinal pathogens.

Diagnosis can be achieved with a faecal flotation method [29]. The vegetative cells found in faeces are colourless [24], long, rod-shaped [88], have a thick laminated double-cell wall [24,29] and a size of 6–8 × 15–20 μm. They can be present alone, in couples or form bonds that might diverge or divide [88]. Typically, they have two vacuoles inside the cytoplasm connected with a bridge, but one or three vacuoles are not uncommon either [24]. Healthy rabbits should have 1–2 vegetative cells per 400× optical field [29]. *C. guttulatus* is considered a pseudoparasite because it closely resembles *Eimeria* oocysts in faeces and can confuse the diagnostician [1]. In addition, the yeast cannot be cultured easily in solid media and has a short life span [23,24].

In the case treatment is required, it usually involves the oral administration of the antifungal compound nystatin [29]. Nystatin has been successfully used in rabbits suffering from diarrhoea [29,87]. Supportive care includes fluid administration and dietary changes, such as carbohydrate restriction and dietary fibre increase [29].

Prevalence can be high for *C. guttulatus* in healthy rabbits, particularly adults. In China, the yeast was found in 83% of rabbits [33]. Cases of *C. guttulatus* infections outside of rabbits [26,29,87] have been described in a cat with diarrhoea [28] and in dogs [30,88,89,90,91,92].

Mixed infections with *P. ambiguus* and *Eimeria* spp. are not uncommon in rabbits. They have been reported in the past, with different prevalence rates, in Greece [93], Germany [37,94,95], Finland [46], Turkey [96], Poland [5,36], Serbia [97], Italy [10], Australia [40], West Africa [98] and Kenya [45]. It is worth noting that in a previous study in Greece, all commercial rabbit farms examined were infected with *P. ambiguus* and *Eimeria* spp. [93]. The presence of such pathogens was not linked to deaths before in our country.

We cannot exclude that the deaths in the current case were not associated with other infectious microorganisms (i.e., *E. coli*), which were not researched. Diarrhoea in rabbits can also result from dietary or environmental causes, which were outside our study’s aim [86]. A gram-stain or culture of faecal material could help rule out other causes of enteritis or dysbiosis [29,31]. However, after administrating the treatments described above for coccidia and *P. ambiguus*, the deaths ceased, rabbits showed clinical improvement, and no more diarrhoea cases were reported. Thus, we can assume that the diarrhoea and deaths were most likely attributed to those parasites. The large numbers of *C. guttulatus* may also have worsened the symptoms [33].

Concerning the sample size used for pooling in both sick and healthy animals (100 faecal samples in each case), research in ruminants indicates that a smaller sample size should also be sufficient in determining the infestation level [99]. A prerequisite for the smaller sample size would be the homogeneous distribution of parasites in each group, that in our case was expected, based on the same symptoms, age and common housing of animals in each group. Alternatively, more pools could have been used to investigate a possible heterogeneity in each group, that in our case was not expected based on the above reasons. However, in our case the pooled samples consisted of 100 samples per group in order to obtain a wider view of the infections.

Finally, it is worth mentioning that anthelmintic resistance in gastrointestinal nematodes is on the rise across different animal species worldwide [98]. To combat this phenomenon, veterinarians should employ selective drug treatment strategies instead of broad-spectrum anthelmintics, such as targeted treatments (T.T.) or targeted selective treatments (T.S.T.) [46,100]. Simultaneously, prophylactic mass treatments of otherwise healthy animals should be limited [100]. The protozoan parasite *Eimeria* spp. has also developed some resistance to different coccidiostats because farmers tend to use them for the chemoprevention of coccidiosis in rabbits [46]. Such is the case for robenidine. It was first introduced in 1982 to replace sulfaquinoxaline/pyrimethamine, which was no longer effective. Initially, robenidine displayed high efficacy against coccidiosis in commercial rabbit farms. However, after four years of constant use, different *Eimeria* spp. slowly developed resistance to the specific drug lowering its efficacy [41]. In addition, robenidine has been shown to reduce litter size and weight gain in young rabbits [101]. Likewise, current research indicates that amprolium and trimethoprim-sulfamethoxazole lack efficacy [43]. On the contrary, sulfadimethoxine, sulfachloropyrazine, toltrazuril, and diclazuril have all recently proven effective in reducing oocyst excretion [43,102] and lesions [43]. Other drugs used in the past, such as salinomycin and maduramycin, appear toxic for rabbits [55]. Veterinarians should also consider the drug withdrawal period when deciding on a treatment regimen to avoid tissue residues [103].

## 4. Conclusions

The current study is a good example of a classic disease in rabbits concerning the simultaneous infection with three common pathogens (coccidia, pinworms and a yeast). A faecal flotation method or other reliable diagnostic technique should be used regularly in industrial rabbit farms for the early detection of gastrointestinal parasites, particularly *Passalurus ambiguus* and *Eimeria* spp. Sample sizes used for pooling should be based on the expected homogenous or heterogenous distribution of parasites inside each animal group, among other factors. Special attention should be given to the numbers of the otherwise apathogenic yeast *Cyniclomyces guttulatus* that can aggravate symptoms in rabbits with gastrointestinal disorders. Consequently, if positive results are obtained, strategic treatments, proper hygiene practices, and sanitisation protocols should be employed. Early diagnosis and control will help to maintain production levels and, therefore, limit financial losses for the farmer while ensuring animal welfare.

## Figures and Tables

**Table 1:** Results of the parasitological analysis using a faecal flotation technique for sick rabbits belonging in Group A (lactating does), Group B (58 days old) and Group C (80 days old). Semi-quantitative assessment was as follows: 1–100 oocysts/eggs/yeast cells per coverslip on a microscopic slide = +; 100–300 oocysts/eggs/yeast cells per coverslip =++; >300 oocysts/eggs/yeast cells per coverslip = + + +.

Groups	Rabbit Age	*P. ambiguus* Eggs	*C. guttulatus* Vegetative Cells	*Eimeria* spp. Oocysts
Group A	Lactating does	++	+++	none
Group B	58 days	+++	+++	none
Group C	80 days	++	+++	+++
